# Safety and immunogenecity of a live attenuated Rift Valley fever vaccine (CL13T) in camels

**DOI:** 10.1186/s12917-016-0775-8

**Published:** 2016-07-26

**Authors:** S. Daouam, F. Ghzal, Y. Naouli, K. O. Tadlaoui, M. M. Ennaji, C. Oura, M. EL Harrak

**Affiliations:** 1 Research and Development Virology, Multi-Chemical Industry, Lot. 157, Z I, Sud-Ouest (ERAC) B.P: 278, Mohammedia, 28810 Morocco; 2 Laboratory of Virology, Hygiene & Microbiology, Faculty of Sciences & Technics, University Hassan II Mohammedia-Casablanca, 20650 Mohammedia, Morocco; 3 School of Veterinary Medicine, University of the West Indies, St. Augustine, Trinidad and Tobago

**Keywords:** Rift Valley fever, Thermostable, Clone 13T vaccine, Camels

## Abstract

**Background:**

Rift Valley fever is an emerging zoonotic viral disease, enzootic and endemic in Africa and the Arabian Peninsula, which poses a significant threat to both human and animal health. The disease is most severe in ruminants causing abortions in pregnant animals, especially sheep animals and high mortality in young populations. High mortality rates and severe clinical manifestation have also been reported among camel populations in Africa, to attend however none of the currently available live vaccines against RVF have been tested for safety and efficacy in this species. In this study, the safety and efficacy (through a neutralizing antibody response) of the thermostable live attenuated RVF CL13T vaccine were evaluated in camels in two different preliminary experiments involving 16 camels, (that 12 camels and 4 pregnant camels).

**Results:**

The study revealed that the CL13T vaccine was safe to use in camels and no abortions or teratogenic effects were observed. The single dose of the vaccine stimulated a strong and long-lasting neutralizing antibody response for up to 12 months.

**Conclusion:**

The presence of neutralization antibodies is likely to correlate with protection; however protection would need to be confirmed by challenge experiments using the virulent RVF virus.

## Background

Rift Valley fever (RVF) is a zoonotic viral disease caused by Rift Valley fever virus (RVFV), which is a virus within the genus *Phlebovirus* and family *Bunyaviridae*. RVF is enzootic and endemic in Africa, Saudi Arabia and Yemen and poses a significant threat to both human and animal health [1-3]. The disease is most severe in sheep, goats and cattle, causing abortions in pregnant females and high mortality in young animals [4-8].

Vaccination is considered to be the most effective way to prevent and control the expansion of the disease. However, the available attenuated vaccines for RVF cause abortions and teratogenic effects (Smithburn strain vaccine) or are thermolabile (CL13 strain vaccine) [6, 9, 10]. An evaluation of efficacy and safety of the CL13 vaccine in ewes at different stages of pregnancy indicated that the vaccine did not induce clinical manifestation of RVF, such as abortion in pregnant ewes, teratogeny in their offspring or pyrexia in vaccinated animals [11]. Vaccination with CL13 vaccine also prevented clinical RVF following virulent challenge. A recent study carried out by Daouam et al. [12] revealed that the CL13 vaccine strain was unstable in both lyophilized and liquid forms at 22-25 °C and at 37 °C, which highlight the importance of cold chain when using the CL13 vaccine in endemic tropical countries. In response to this need, the CL13 vaccine strain was made thermostable through three cycles of heating (56 °C) and selection of thermostable particles (12). The thermostable vaccine clone (CL13T) was found to be safe when tested on sheep, goats and cattle with no clinical signs or side effects observed. Neutralizing antibodies were detected in vaccinated animals for a minimum of one year in sheep and goats, and for at least 4 months in cattle indicating that the vaccine also provide a good protection [12].

Recent outbreaks of RVF in western Africa have resulted in high mortality and severe clinical signs among dromedary camel populations [13]. Serological surveys in northern and eastern Africa have revealed high percentages of seropositive camels after the occurrence of an outbreak [14, 15]. Camels are therefore susceptible to RVFV, and are likely to play a role in the epidemiology of the disease and should be included in any vaccination program. There have been no previous reports of camels being vaccinated against RVF, and no RVF vaccines including a thermostablized vaccine have been tested for safety and efficacy in this species. This study therefore was conducted to evaluate the safety and efficacy of the thermostable live attenuated CL13T vaccine in camels.

## Methods

### Vaccine (CL13T) production and titration

The CL13T attenuated virus was propagated and titrated on African Green monkey kidney cells (Vero, ATCC) [9]. The vaccine was formulated by mixing V/V the viral suspension with a stabilizer of lyophilisation (4 % peptone, 8 % sucrose and 2 % glutamate). The viral suspension titre is fixed to have a minimum of 10^6^ DICT_50_ per dose. The RVF CL13T vaccine was distributed in glass vials and freeze dried. Before administration, the vaccine was reconstituted by adding a saline solution.

### Safety and efficacy testing of the CL13T candidate vaccine in camels

All experiments involving camels (*Camelus Dromedarius*) were carried out in accordance with guidelines for the care and handling of experimental animals these animals are dedicated to research and maintained an experimental farm. The animal experiments were approved by the MCI ethics committee in charge of the control and supervision of experiments on animals and the experiments were conducted in high containment level 3 facilities. Sixteen dromedary’s camels 2-3 year old, negative for RVFV antibody by the neutralization test were divided in two groups of six and one group of 4.

In experiment 1: Two groups of 6 camels were vaccinated subcutaneously (SC) with a dose of 10^6^ TCID_50_ of the CL13T vaccine. Camels in group 1 received a single dose of the vaccine and camels of group 2 received a booster dose, four weeks after the first vaccination. The general behavior and rectal temperature of the camels were recorded daily for two weeks after each vaccination as well as observation for local inflammation at the injection site. Sera samples were obtained daily for the first 15 days post-vaccination (PV) and tested for RVFV RNA and infectious virus by quantitative real-time PCR [16] and virus isolation in Vero cells, respectively. Antibody titers were monitored over a 12 months period by virus-neutralization (VN) on Vero cell culture as described in the OIE manual (OIE, 2012). VN was performed on 96 wells plates using 100 DL_50_ of the RVFV (CL13) per well with a serial 3 fold dilution of the serum in a 4 wells replicate foe each dilution. After 60 mn of neutralization at 37 °C, the cells suspension was added and the plate incubated 5 days before reading. The neutralizing titre is determined by Reed et Muench method and expressed in log10/ml. Sera were also tested for IgG detection by a competitive ELISA (ID-VET RIFTC-4P).

In experiment 2: a group of 4 pregnant she camels were vaccinated via the SC route with a 10X the normal dose (10^7^ TCID_50_) of the CL13T vaccine. Rectal temperatures, appetite and behavior of each animal were documented daily for 1 month and then every month during five months after vaccination until calving for a potential abortions effect and teratogenicity.

### Statistical analysis

The antibody titers detected in the first and second group of camels were compared using the Student *t* test; with a significance level of *p* = 0.05.

## Results

### Safety testing of CL13T candidate vaccine in camels

The C13T vaccine was found to be safe, with no evidence of abortions or teratogenicity among the offsprings of the vaccinated pregnant camels. All camels were healthy and did not have any sign of illness. Normal body temperatures were recorded in the pregnant as well as among the camels in Group 1 and 2 before vaccination and no local reactions were recorded at the injection sites. In the 15 days after vaccination no abnormal behavior was observed in any of the vaccinated animals and their body temperatures remained in the normal range. Very low levels of viral RNA (Cycle Threshold values from 37.6 to 38.6 among a total of 40 cycles) were detected in the blood of 7 of the camels in Groups 1 and 2 during the first 2 weeks following vaccination. However, no infectious virus was isolated from the samples after 2 blind passages on Vero cells. The absence of RVFV in the inoculated cells was confirmed by qPCR.

### Serological responses in camels vaccinated with the CL13T candidate vaccine virus

**Fig. 1:**
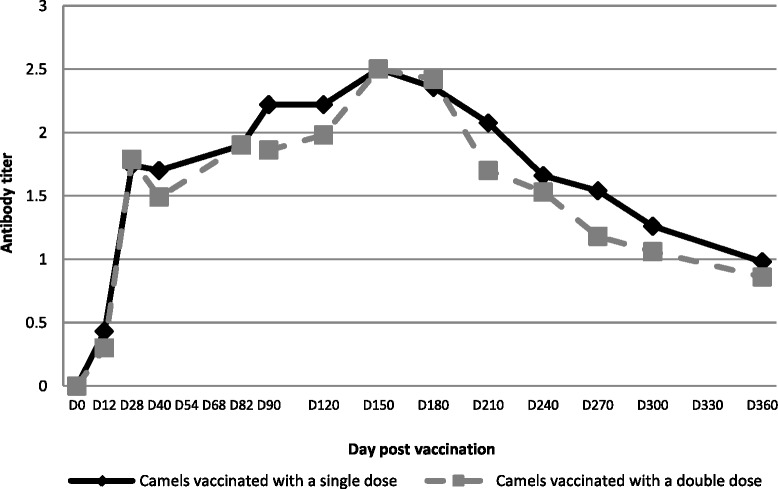
Neutralizing antibody titres in camels vaccinated with a single and a double dose of live CL13T RVF vaccine. All camels were vaccinated subcutaneously (SC) with a dose of 10^6^TCID_50_ of the CL13T vaccine. Camels in group 1 received a single dose and camels of group 2 received a booster at day 30 after vaccination

Neutralizing antibody were recorded in all the vaccinated camels by day 12 PV, with peak neutralizing titers of 2.5 log DN_50_ (equivalent to a serum dilution of 1:500) being observed on day 28 (PV) (Fig. 1). High titers of neutralizing antibody were maintained for a period of 6 months PV, at which time the titers started to wane over the next 6 months, reaching at titer of 0.92 log DN_50_ (equivalent to a serum dilution of 1:10) at twelve months post-vaccination (Fig. 1). Similar antibody titers were detected in camels vaccinated once (group 1) and twice (group 2), showing that there was no significant increase in neutralizing antibody titers through the administration of a booster dose of the vaccine.

**Fig. 2:**
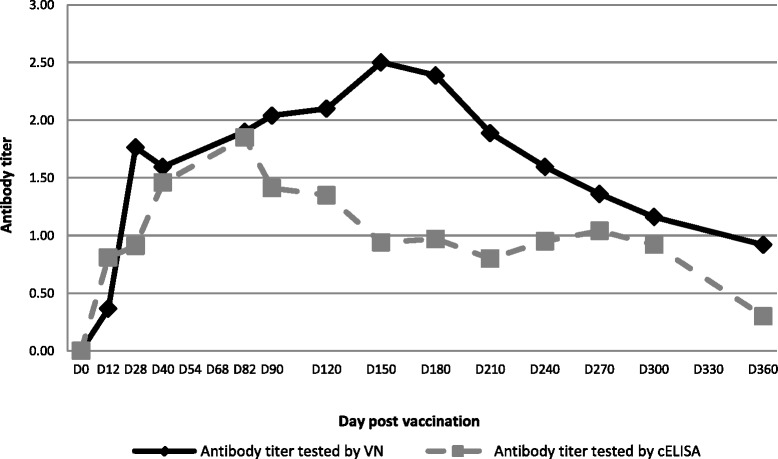
Antibody titres of camels vaccinated with a live CL13T RVF vaccine tested by VN and cELISA. Neutralizing antibody were tested in all vaccinated camels by VN and cELISA test, a significant differences (*p* < 0.05) in antibody titers were observed

Significant differences (p < 0.05) in antibody titers were observed in the sera samples from camels tested by VN as compared to those tested by cELISA. Antibody titers measured by the two tests (VN and cELISA) remained similar for the first 3 months post-vaccination and then diverged to attain titers that were significantly different (Fig. 2). Results revealed a reduced sensitivity of the cELISA compared to the VN test for the detection of RVFV antibody (Fig. 2). It is important to note however that the cELISA kit used in this study has only been validated for use in ruminants. Thus, results indicate that this cELISA may not be optimized for use in camels and that the sensitivity of the assay may need to be improved before it can be recommended for routine diagnosis or for vaccination monitoring in camels.

## Discussion

This study reveals that camels mounted a strong and long-lasting neutralizing antibody response when vaccinated with a single dose of the live CL13T RVF vaccine and that the vaccine is safe to use, producing no significant side-effects in the vaccinated animals. The neutralizing antibody response was similar to that observed in small ruminants and cattle after vaccination with the live RVF clone 13 vaccine [17, 18].

This is the first report that evaluates the safety and efficacy of a live attenuated thermostabilized RVF vaccine in camels. To be sure that this vaccine protects camels against RVF, challenge studies would need to be carried out. However in the case of RVF, neutralizing antibodies are known to be reliable predictor of protection after vaccination [17, 19]. The high levels of neutralizing antibodies observed in the vaccinated camels may indicate therefore that the CL13T vaccine is likely to be protective in camels for up to 12 months.

It is highly recommended to vaccinate livestock to prevent the occurrence of disease in susceptible animals and if possible virus amplifying hosts, in order to break the epidemiological transmission cycle. With the high densities of dromedary camels in many areas where RVF is present, it is extremely important to be able to protect camels, as well as other susceptible species.

This is the first study that has evaluated the safety and immunogenicity of a live attenuated RVF vaccine in camels. A recent study, reported camel vaccination with a recombinant adenovirus encoding RVFV envelope glycoprotein. A lower antibody neutralizing titre was observed in camels comparatively to other species (sheep, goats and cattle) with a dose of 10^9^ injected intramuscularly in the presence of an adjuvant [19]. No challenge was carried out for the protection testing.

## Conclusions

In our study, the obtained antibody response in vaccinated camels was similar to that registered in sheep and better than cattle response with a dose of 10^6^ only, injected by S/C route. Although this is a preliminary study, the presence of neutralization antibodies is likely to correlate with protection. To be noted that RVF protection of camels has never been tested by experimental infection and no challenge model has been developed for camels.

In summary, the study showed that the CL13T live attenuated vaccine is safe to use in camels and that a single dose of the vaccine stimulated strong and long-lasting neutralizing antibody response for up to 12 months. In the absence of any tested live vaccine for camels, the CL13T vaccine could be considered for camel protection against RVF after a field trial that include sufficient number of animals under the normal living conditions.

## Abbreviation

CL13, clone 13; CL13T, clone 13 thermostable; PV, post-vaccination; RVF, Rift Valley fever; RVFV, Rift Valley fever virus; SC, subcutaneously; Vero, African Green monkey kidney cells; VN, virus-neutralization
